# Recent advances in understanding/management of premenstrual dysphoric disorder/premenstrual syndrome

**DOI:** 10.12703/r/11-11

**Published:** 2022-04-28

**Authors:** Lara Tiranini, Rossella E Nappi

**Affiliations:** 1 Research Center for Reproductive Medicine, Gynecological Endocrinology and Menopause, IRCCS San Matteo Foundation, Pavia, Italy; 2 Department of Clinical, Surgical, Diagnostic and Pediatric Sciences, University of Pavia, Pavia, Italy

**Keywords:** Premenstrual syndrome, premenstrual dysphoric disorder, progesterone, allopregnanolone, neurosteroids, depression, inflammation, therapeutic strategies, prevention

## Abstract

Premenstrual syndrome (PMS) and premenstrual dysphoric disorder (PMDD) are common disorders of the luteal phase of the menstrual cycle and are characterized by moderate to severe physical, affective, or behavioral symptoms that impair daily activities and quality of life. PMS and PMDD have recently raised great interest in the research community for their considerable global prevalence. The etiology of PMS/PMDD is complex. Ovarian reproductive steroids (estradiol and progesterone) are considered pathogenetic effectors, but the key feature seems to be an altered sensitivity of the GABAergic central inhibitory system to allopregnanolone, a neurosteroid derived from progesterone produced after ovulation. Also, a reduced availability of serotonin seems to be involved. New insights point to a role for genetic and epigenetic modifications of hormonal and neurotransmitter pathways, and inflammation is the potential link between peripheral and neurological integrated responses to stressors. Thus, new therapeutic approaches to PMS/PMDD include inhibition of progesterone receptors in the brain (i.e., with ulipristal acetate), reduced conversion of progesterone to its metabolite allopregnanolone with dutasteride, and possible modulation of the action of allopregnanolone on the brain GABAergic system with sepranolone. Further research is needed to better understand the interaction between peripheral inflammatory molecules (cytokines, interleukins, C-reactive protein, and reactive oxygen species) and the brain neurotransmitter systems in women with PMS/PMDD. If confirmed, neuroinflammation could lead both to develop targeted anti-inflammatory therapies and to define prevention strategies for the associated chronic inflammatory risk in PMS/PMDD. Finally, the observed association between premenstrual disorders and psychological diseases may guide prompt and adequate interventions to achieve a better quality of life.

## Introduction

Premenstrual syndrome (PMS) is a common disorder in women of reproductive age and is characterized by at least one physical, emotional, or behavioral symptom, which appears in the luteal phase of the menstrual cycle and resolves shortly after the onset of menses^1^. The spectrum of symptoms is wide and the most common are breast tenderness, bloating, headache, mood swings, depression, anxiety, anger, and irritability. They must interfere with daily personal and occupational life during two menstrual cycles of prospective recording^2,3^.

The most severe form of PMS is defined as premenstrual dysphoric disorder (PMDD), characterized predominantly by emotional and affective symptoms not due to another psychiatric condition^4^. PMDD was included as a new diagnostic category of depressive disorders in the Diagnostic and Statistical Manual of Mental Disorders (DSM-5)^5,6^ and recently (2019) coded as a gynecological diagnosis in the World Health Organization’s International Classification of Diseases (ICD-11)^7^. A PMDD diagnosis requires the presence of at least one mood symptom (marked affective lability, irritability, depressed mood, anxiety, or tension) in a group of at least five (including loss of interest, subjective difficulty in concentrating, fatigue, marked appetite change with overeating or food cravings, insomnia or hypersomnia, feeling emotionally overwhelmed, and physical symptoms). Such symptoms should occur during the luteal phase of the majority of menstrual cycles over the previous year. Furthermore, they must be associated with clinically significant distress regarding social, academic, or working activities; they should not be the exacerbation of a chronic condition or the effect of medications, and they need to be confirmed by prospective daily ratings during at least two symptomatic cycles^6^.

According to a recent meta-analysis, premenstrual symptoms are very common, affecting about half of women of reproductive age worldwide^8^. However, prevalence rates widely vary in different studies and countries depending on diagnostic criteria and methods of investigation. PMS is estimated to affect 20 to 30% of women in the United States^2^, a minimum of 12% in France^9^, and a maximum of 98% in Iran^10^, while PMDD ranges from 3 to 8% of women in the United States^11^ to 17% in Brazil^12^. The estimation of the prevalence of PMS/PMDD in different countries is becoming increasingly important^13^, and the focus should be on women of a younger age to raise awareness and improve management^14–16^.

Moderate to severe PMS and PMDD significantly reduce quality of life^17^ and raise societal costs associated with decreased work productivity, work absenteeism, and increased use of healthcare services^18,19^. That being so, research on PMS/PMDD is of paramount importance and pinpoints etiology and co-occurring conditions as well as appropriate available treatments and potential new therapeutics.

## Neurotransmitters, hormones, and neurosteroids

According to the complex pathophysiology of moderate to severe PMS and PMDD that predominantly involves central neurotransmitters, ovarian hormones, and neurosteroids^20^, the main therapeutic approaches target both the brain neurotransmitter systems and the hypothalamus-pituitary-ovarian axis. A brief revision of the currently available first-line treatments is mandatory to introduce new insights and the latest therapeutic proposals.

### First-line treatments

Currently, the first-line treatment for PMDD consists of selective serotonin reuptake inhibitors (SSRIs), such as fluoxetine, paroxetine, sertraline, and escitalopram^21^. Serotonin is a pivotal neurotransmitter modulating mood and behavior. It plays a fundamental role in the pathophysiology of PMS/PMDD because women with the condition have atypical serotonergic transmission, a lower density of serotonin transporter receptors, decreased plasmatic serotonin levels in the luteal phase, and higher serotonin responsiveness in the follicular rather than in the luteal phase^22^. Moreover, ovarian sex steroids, acting on the monoamine oxidase (MAO) enzyme responsible for serotonin degradation^20^, influence serotonin brain availability. SSRIs usually require 4 to 8 weeks to reach clinical efficacy in the treatment of depression and anxiety disorders. By contrast, their effect on severe PMS/PMDD is rapid: within a few days to a maximum of 4 weeks since the start of treatment^23,24^. Thus, besides a continuous administration throughout the menstrual cycle, SSRI regimens may be intermittent, starting about 14 days prior to the expected menstruation (i.e., during the luteal phase) or even at the onset of severe PMS/PMDD symptoms and discontinuing at menses^21,25,26^. Moreover, SSRIs may be administered in a semi-intermittent regimen that consists of a low SSRI dose during the follicular phase and a higher SSRI dose during the luteal phase^25^. According to a Cochrane review, SSRIs are effective in reducing symptoms of PMS/PMDD whether used continuously or intermittently^25^. Usually, side effects of SSRIs compromise patient compliance^27^; then, intermittent regimens offer the advantage of better tolerability with a higher grade of acceptability and a lower incidence of pharmacological dependence^28^.

Combined hormonal contraception (CHC) may represent an alternative to treatment. The rationale for CHC is the blockade of an ovulatory surge of sex steroids since premenstrual symptoms are not observed during anovulatory cycles^29^ and disappear when women undergo treatment with agonists of gonadotropin-releasing hormone (GnRH)^30^ or bilateral oophorectomy^31^. The most effective CHC is a combination of the progestogen drospirenone and ethinyl estradiol in a regimen with a shorter hormone-free interval (4 rather than 7 days)^32,33^. Nevertheless, CHC may lead to side effects, including deterioration of mood, especially in vulnerable women (i.e., those with a previous diagnosis of mood, anxiety, or eating disorders)^34^. Moreover, CHC is not an appropriate option for women who are planning a pregnancy.

### Progesterone and selective progesterone receptor modulator

Converging evidence suggests that fluctuations of ovarian sex steroids (in particular, progesterone) are key factors for PMS/PMDD^35^, given the synchrony with the post-ovulatory phase and the reinstatement of symptoms during GnRH agonist treatment when add-back progesterone is administered^36^. Since women with PMDD have progesterone serum concentrations similar to those of healthy women^37^, the underlying mechanism of PMDD is presumed to be an increased sensitivity to fluctuations of this steroid^38,39^. Progesterone interacts with the chemistry of the central nervous system (CNS)^40–42^ by easily passing through the blood-brain barrier. Progesterone receptors (PRs) are indeed widespread in the amygdala, hippocampus, hypothalamus, and frontal cortex^43,44^. Back in the early 1990s, it seemed biologically plausible that selective progesterone receptor modulators (SPRMs) represent a treatment of PMDD because of their antagonistic action on PRs. The first drug investigated, mifepristone, failed to improve symptoms of severe PMS. Clinical trials were randomized but displayed some limitations in the study designs^45,46^. More recently, ulipristal acetate (UPA), a second-generation SPRM already employed for emergency contraception and for the treatment of uterine fibroids^47,48^, was tested as a suitable option at low chronic dosing (5 mg/day) to ameliorate symptoms in women with PMDD. The first proof-of-concept randomized controlled trial on UPA showed improvement in emotional and behavioral symptoms of PMDD^49^. However, whether the effect of UPA was mediated by induction of anovulation or by specific actions on PR could not be established. UPA is currently considered a promising drug in the management of PMDD. Belonging to a class of compounds that fulfill the goals of precision medicine, UPA can be an alternative pharmacological treatment when antidepressants are not tolerated or are poorly beneficial^49,50^.

### GABAergic system and therapies

Extensive research demonstrates that the central effects of progesterone on mood result largely from its metabolite allopregnanolone, a neuroactive steroid that acts as a strong positive modulator of the gamma-aminobutyric acid (GABA) receptor^51–53^. GABA is the main inhibitory neurotransmitter within the CNS and is a pivotal regulator of stress, anxiety, vigilance, and seizures^37^. The involvement of the GABAergic system in the pathophysiology of PMS/PMDD has recently aroused growing interest in finding new therapies directly focused on premenstrual symptoms. At high concentrations, allopregnanolone can cause sedation by activating the GABA receptor, but it may also induce paradoxical reactions with adverse moods in susceptible women^54^. Those with severe PMS/PMDD have normal levels of plasma allopregnanolone^41,55–57^, but some evidence showed diminished concentrations of allopregnanolone and its precursor progesterone and a blunted response to the GnRH test during the luteal phase of the menstrual cycle^58^. Fluctuations of allopregnanolone induce changes in the conformation of the GABA-A receptor sufficient to determine anxiety-like behaviors in predisposed women^59,60^. In light of these findings, the development of new treatments for PMDD attempted to stabilize allopregnanolone signaling^50^. Dutasteride, an inhibitor of the enzyme 5alpha-reductase that converts progesterone to allopregnanolone, was recently tested with the aim of modulating progesterone/allopregnanolone balance in women with PMDD. Dutasteride prevented the luteal phase increase in allopregnanolone and improved most PMDD symptoms (i.e., irritability, anxiety, sadness, food cravings, and bloating) without exerting any effect on healthy controls^61^. At present, dutasteride is a potential off-label option for women experiencing side effects or lacking benefits of SSRIs^61^.

Given the plasticity of the GABA-A receptor (namely changes in subunit composition and pharmacological properties) in response to allopregnanolone in predisposed women^53,62^, the blockade of allopregnanolone action on the GABA-A receptor has been investigated as a possible treatment of PMDD. Allopregnanolone effects can be antagonized by its endogenous isomer isoallopregnanolone, a GABA-A receptor modulating steroid antagonist (GAMSA)^63^. In an explorative first-in-man phase IIa treatment study, women with PMDD who received isoallopregnanolone (sepranolone, UC1010) subcutaneously every second day during the luteal phase have shown a significant reduction in PMDD symptoms compared with placebo^64^. The treatment was effective in reducing mood symptoms and impairment; no statistically significant effects have been detected on the physical symptoms, and no safety concerns were identified^64^. In a subsequent phase IIb randomized, double-blind, placebo-controlled study using sepranolone in two dosages (10 mg or 16 mg)^65^, the placebo effect was 30% higher compared with the phase IIa study^64^. However, an attenuating effect by sepranolone on mood symptoms, impairment, and distress in PMDD women was demonstrated, while no significant improvement on physical symptoms was noted compared with placebo^65^. Interestingly, the higher dosage (16 mg) of sepranolone appeared less effective than the 10-mg dose, allowing the speculative hypothesis of a possible biphasic dosage-response effect on mood as shown for allopregnanolone plasma concentrations^61,66,67^. Therefore, further research is needed to determine whether isoallopregnanolone, which antagonizes the effect of allopregnanolone on the GABA-A receptor, has the potential to become a treatment for PMDD^64,65^. The US Food and Drug Administration recently approved allopregnanolone itself (brexanolone) for the treatment of postpartum depression (PPD)^68,69^, a disorder extensively associated with PMS/PMDD^70,71^ in the context of reproductive depression^72^. Exposure to high allopregnanolone levels during pregnancy has a protective and mood-stabilizing effect, while in susceptible women, the sudden decrease in allopregnanolone following placental detachment at birth alters the GABAergic signaling^73–76^. Similarly, other positive allosteric modulators of the GABA-A receptor represent potential new drugs for PPD^77^. For instance, zuranolone and ganaxolone are neuroactive steroids currently being evaluated in trials^78,79^. To further elucidate the apparently discrepant effects of allopregnanolone in PMDD compared with PPD, considering women with both disorders, including genetic and epigenetic measures will be mandatory in future research in order to identify risk markers for allopregnanolone sensitivity^35^.

## Genetics

Certain genetic variations may predispose to the development of PMDD. The first positive genetic finding dates back to 2007 when an association between PMDD and variants of estrogen receptor 1 (ESR1) was demonstrated^80^. More recently, Dubey *et al*.^81^ found that genes of the estrogen-sensitive epigenetic ESC/E(Z) complex are expressed differentially in lymphoblastoid cell lines isolated from women with PMDD compared with healthy controls. This gene family is an effector of response to ovarian sex steroids and acts as a gene silencing network through epigenetics, the ultimate mechanism for translating environmental signals into permanent changes in gene expression^50^. Very recent findings revealed the molecular mechanism underlying the different cellular response to estradiol observed in women with PMDD^82^. Indeed, consistent with the transcriptome comparison between PMDD and healthy women, a blunted intracellular endoplasmic reticulum stress response and an altered calcium homeostasis were found in women with PMDD, thus suggesting an increasing neuronal excitability resistant to GABA-A receptor modulators^82^.

In a mouse model, a single-nucleotide polymorphism in the brain-derived neurotrophic factor (BDNF) gene induces anxiety-like and depression-like behavior in response to estradiol administration, similarly to what occurs in women with PMDD^83^.

Finally, recent findings focused on genetic variations involving the GABAergic system, establishing for the first time an association between PMDD and copy number variations in the *GABRB2* gene encoding for a GABA-A receptor subunit^84^. Thus, genetic and epigenetic studies may shed light on a possible behavioral sensitivity to ovarian sex steroids and may pave the way to novel targets for therapy^83^.

## Inflammation

There is emerging interest in determining whether exaggerated immune-inflammatory response contributes to PMS/PMDD^85,86^. Estradiol and progesterone have anti-inflammatory and anti-oxidative properties^86^ and their decline in the late luteal phase leads to increased endometrial oxidative stress and synthesis of pro-inflammatory prostaglandins, cytokines, chemokines, and matrix metalloproteinases^87,88^. Extensive research has already linked chronic inflammation to psychiatric and somatic disorders having common features with severe PMS/PMDD, including depression, anxiety, migraine, and chronic fatigue syndrome^89–93^. Thus, in recent years, numerous studies have investigated a possible association between peripheral inflammation and PMS/PMDD, even though the results seem controversial.

Peripheral levels of pro-inflammatory interleukins and tumor necrosis factor-alpha (TNF-α) were found to be elevated in women with PMS^94^. Levels of C-reactive protein (CRP), another biomarker of inflammation, were positively related with PMS symptom severity, especially mood, behavior, and pain symptomatology^95,96^, but new studies highlight the absence of a significant increase in peripheral CRP levels in women with PMS^97,98^. Moreover, a rise in the acute-phase protein haptoglobin and in plasma complement C3 and C4 was observed, albeit not in the inflammatory range^97^. Interestingly, gut microbiota varies during the menstrual cycle and according to the severity of premenstrual symptoms^99^.

Studies examining the presence of oxidative stress levels in women with PMS are scarce and ultimately reach conflicting conclusions. Oxidative stress did not seem to be increased in women with PMS^97,100^, whereas previously, Duvan *et al*.^101^ showed a reduced plasma antioxidant capacity in the luteal phase of the menstrual cycle in women with PMS. These new findings were confirmed in an up-to-date systematic review^102^. Moreover, a recent prospective study showed that serum concentrations of antioxidant vitamins A, C, and E were generally not associated with PMS symptoms or severity, supporting the evidence that the use of antioxidant vitamins as a remedy for PMS may be inconclusive^103^. On the other hand, zinc supplementation manifested different properties on inflammation and premenstrual symptoms. Indeed, it seemed to improve premenstrual symptoms and total antioxidant capacity in women with PMS/PMDD^104^. In addition, it increased levels of BDNF^104^, a known regulator of neurogenesis influenced by sex steroids and whose levels are reduced in women with PMS^105^. Interestingly, zinc displays multiple beneficial effects, including antioxidant, anti-inflammatory^106^, and antidepressant^107^ actions, and its role as a PMS modulator may manifest through an inhibition of extrasynaptic GABA-A receptors^108^.

Recent research focused also on chemokines, which have already been associated with generalized anxiety disorder^109^, chronic stress^110^, and food intake^111^. Some chemokines (CCL2, CCL5, and CCL11) predicted more severe PMS symptoms, thus underlying a possible link between uterus and brain function through the uterine-chemokine-brain axis^112^.

Increasing evidence points to neuroinflammation expressed via the GABAergic system as an etiological factor for PMS/PMDD^113^. Research in humans is limited and the majority of data are obtained from animal studies. It is observed that positive allosteric modulators of the GABA-A receptor, such as allopregnanolone, attenuate the impact of inflammation in animal models but that inhibitors of GABA-A receptor activity increase pro-inflammatory responses^114–116^. In this context, women with PMDD whose GABA-A receptor plasticity and sensitivity are altered manifest an unexpected GABAergic response to allosteric modulators and subsequently show opposite effects on neuroinflammation compared with healthy subjects^117^.

Because the GABA-A receptor channel is permeable to chloride, an altered neuronal chloride homeostasis is a possible contributor to the paradoxical GABAergic response to allopregnanolone observed in vulnerable adults^67,118^. Cation-chloride co-transporters control the intracellular chloride gradient across neurons^119^. The Na-K-2Cl co-transporter (NKCCl) that mediates chloride influx and the K-Cl co-transporter isoform 2 (KCC2) that regulates chloride efflux is the most relevant^119,120^. In the normal adult CNS, the outward-directed pump KCC2 dominates, thus maintaining a low intracellular chloride concentration. Therefore, the activation of the GABA-A receptor triggers chloride influx, causing hyperpolarizing inhibition^119^. Indeed, via ligand-gated GABA-A receptor channels, GABA has a general hyperpolarizing action and an inhibitory role in adult neurons^121^. Conversely, in neurological conditions such as seizures, neuropathic pain^118,122^, or peripheral inflammation^123^, microglia and sensory fibers secrete BDNF that binds to tyrosine kinase B (TrkB) receptors on neurons and triggers a downregulation of KCC2 pump^119^. The result is a higher intracellular chloride concentration that leads to a reversed polarity of GABAergic neurotransmission, causing GABA to become depolarizing and excitatory^67,119^. According to these findings, genetic mutations affecting chloride co-transporter functions have been associated with anxiety-like behaviors and other neurological conditions in mice^124^. Interestingly, estrogens modulate the GABAergic tone through action on chloride homeostasis^121,125^. More specifically, estradiol enhances the activity of NKCCl with a subsequent increase in intracellular chloride concentration^121,126^. Thus, the GABA-A receptor action causes chloride efflux, resulting in depolarization and hyperexcitation^121^. This is in line with evidence suggesting that increasing levels of estradiol during the luteal phase of the menstrual cycle seem to provoke more negative mood symptoms^127,128^. Therefore, chloride intracellular concentration has to be taken into account to guide treatments involving GABA-A receptor-modulating agents^118,129^.

As far as inflammation is concerned, the GABAergic system plays a major role in modulating the biological stress response^113^. Animal studies revealed that the administration of allopregnanolone normalizes hypothalamus-pituitary-adrenal (HPA) axis dysfunction through the sedative properties of enhanced GABAergic transmission^130^. Moreover, allopregnanolone improves the hippocampal neurogenesis affected by chronic stress^131^. According to a study by Girdler *et al*.^132^, acute stress increases levels of allopregnanolone with inhibitory effects in healthy women, whereas those with PMDD do not exhibit the typical allopregnanolone surge. Moreover, chronic stress has been shown to alter GABA-A receptor subunit composition and sensitivity to modulators^67,133,134^. Indeed, women with PMDD perceived daily events as more stressful and reacted to stressors with higher arousal of negative feelings when compared with controls, whereas a delayed and blunted HPA function was observed as in other stress-related conditions^135^.

However, wider research and prospective studies are needed to determine the etiological relationship between inflammation, HPA axis, and neurosteroidal modulation of GABAergic function in PMS/PMDD in order to determine whether treatments targeting inflammatory pathways could improve symptom severity and quality of life^85^. Moreover, the identification of a causal relationship between chronic inflammation and PMS/PMDD will enable us to consider this reproductive disorder a sentinel of future chronic disease risk, given the evidence that women with PMS have a higher risk of developing hypertension^136^.

## Other trends in research

In recent years, increasing research further investigated the comorbidities of severe PMS/PMDD, and a strong association with psychiatric disorders was confirmed^12,137^. Women with severe PMS/PMDD are at higher risk to develop PPD^70^ and suicide experiences^138–140^ and manifest increased incidences of generalized anxiety disorder^141^, bipolar disorder^142^, eating disorders^143^, addictive behaviors such as nicotine or alcohol use^144^, and poor sleep quality^145^. Exposure to traumatic events, childhood physical and emotional abuse, and post-traumatic stress disorder also correlate with PMS/PMDD^146–148^. Moreover, personality traits, especially neuroticism, and negative attitudes toward menstruation cause dysfunctional coping and maladaptation to physiological menstrual cycle changes, thus determining distress and functional impairment^137,149,150^. Based on these findings, identifying behavioral and cognitive features associated with PMS/PMDD is of utmost importance in order to provide the proper treatment to improve quality of life^12^. A recent systematic literature review outlined that psychoeducation and cognitive behavioral therapy (CBT) are effective in ameliorating PMS/PMDD^151^. More specifically, mild to moderate PMS could benefit from relaxation techniques and psychoeducation, while severe PMS and PMDD required one-to-one CBT^151^. According to this review, CBT proved successful in treating PMS mood symptoms of varying severity in a population of young women^152^. Furthermore, the first internet-based CBT trial proved to be highly effective in reducing PMDD^153^. Also, regular exercise appeared to be effective in relieving both physical and psychological symptoms of mild to severe PMS^154,155^. However, more studies are warranted to compare the impact of different types of physical activities. In particular, taking into account symptom severity by distinguishing between PMS and PMDD according to validated criteria is essential^6^. That being so, evidence supports the possibility of recommending non-pharmacological treatments (CBT and lifestyle modifications) as part of a personalized treatment plan for mild to severe PMS and PMDD. Finally, herbal (*Vitex agnus-castus*)^156,157^ and some complementary (vitamins, calcium, and magnesium)^158–161^ therapies have been investigated in randomized controlled trials. Future meta-analyses will evaluate their efficacy as treatments for milder forms of PMS.

## Summary and Conclusions

The pathogenesis of PMS/PMDD is complex and multifaceted. According to recent research, a key etiological role is played by altered sensitivity of the GABAergic central inhibitory system to allopregnanolone. Genetic and epigenetic susceptibility may contribute, and inflammation may represent the link between the peripheral and the integrated neurological response to stressors. Thus, the new therapeutic approach to severe PMS/PMDD targets the brain neurotransmitter systems through modulation of the action of allopregnanolone on the GABA receptor. More research is needed to better understand the role of neuroinflammation in order to both develop targeted anti-inflammatory therapies and define prevention strategies for the associated chronic inflammatory risk. Finally, the characterization of PMS/PMDD as a major indicator for other comorbid diseases could allow prompt and adequate intervention to safeguard the quality of life.

## Abbreviations

BDNF, brain-derived neurotrophic factor; CBT, cognitive behavioral therapy; CHC, combined hormonal contraception; CNS, central nervous system; CRP, C-reactive protein; GABA, gamma-aminobutyric acid; GnRH, gonadotropin-releasing hormone; HPA, hypothalamic-pituitary-adrenal; KCC2, K-Cl co-transporter isoform 2; NKCCl, Na-K-2Cl co-transporter; PMDD, premenstrual dysphoric disorder; PMS, premenstrual syndrome; PPD, postpartum depression; PR, progesterone receptor; SPRM, selective progesterone receptor modulator; SSRI, selective serotonin reuptake inhibitor; UPA, ulipristal acetate
